# Sterilizing Sponge in Pulp Capping

**Published:** 1892-12

**Authors:** T. H. Paramore

**Affiliations:** Hampton, Va.


ARTICLE V.

### STERILIZING SPONGE IN PULP CAPPING.

BY T. H. PARAMORE, HAMPTON, VA.

Read before the Southern Dental Association.

In a paper read before the meeting at Old Point, Va., of the Southern Dental Association, in August, 1887, I took this position: "Irritation and inflammation consequent upon a wound, is an effort upon the part of nature to repair the injury; the surgeon or dentist who renders nature greatest assistance in her effort at repair, comes nearest the ideal towards which we are struggling."

These views, the success attained in general surgery in the use of sterilized sponge, and a belief that nodules of asteo-dentine are unsuccessful attempts of the pulp to protect itself against decay, induced me to undertake the preservation of the life of an exposed pulp, a practice I had abandoned.

It is not my purpose in this paper to state the process of reasoning by which I arrived at my present mode of treatment. The idea in treatment is to place at point of pulp exposed a material that will catch and hold the bone forming product of the pulp so that it may form where it will protect, and not, as is often the case, in the body of the pulp, where it irritates.

As my friend, Dr. Wright, so aptly expounds it, "The sponge is the staging used by the pulp in erecting its fortifications." For more than six years I have been using sterilized sponge for this purpose with a success far greater than I at first dared hope. The following is my method of operating:

I assume that the cavity is prepared, the dam adjusted and the point or pulp-exposure clearly defined and visible. First, thoroughly remove all foreign matter from cavity.

2d. Cleanse perfectly with 1-509 per cent. solution of bichloride of mercury, fingers, dam, cavity instruments —everything that, directly or remotely, comes in contact with cavity. I use bibulous paper for this purpose.

The following preparation will be found very convenient, it does not deteriorate with age:

℞

| | |
|---|---|
| Acid Hydrochlorici - - - | ʒ i |
| Hydr. Bichloride, - - - - | ʒ i |
| Alcoholic, - - - - - | ℥ i |

M. Sig: 10 drops to one ounce, 1-500 per cent. solution.

3d. Hold the sponge, wrapped in oil silk, (never allow it to come in contact with the fingers) in the left hand, and with plyers tear off a piece the required size, place this upon the pulp at the point of exposure. This is, at times, very difficult to accomplish; it is sometimes necessary to dampen the cavity with bichloride solution, which renders it much easier.

4th. Fill with oxy-phosphate in the usual manner, being careful not to dislodge the sponge. The tooth often aches for some time after the operation, but don't disturb it; if the operation has been properly performed the tooth will get easy, and the pulp will not die.

For some time after I began this treatment, I allowed the oxyphosphate to remain in for several months, removed all, or part, of the oxy-phosphate, and filled permanently; but now, unless there is good reason for acting otherwise, I fill permanently immediately, or in a few days.

In quite a number of cases treated as described, I have found after some months the point of pulp-exposure covered and securely sealed by osteo-dentine, and the tooth alive and well.

In one hundred and fifty cases that I have been able to keep trace of, I have a record of twenty-three failures. I have a bicuspid filled as described in 1886, upon the anterior surface. In December, 1891, this tooth began to ache by nerve exposure in a crown cavity. I will send this tooth to your honored president that you may all see how perfectly the pulp is protected on anterior surface.

I find this record of the case: October, '86, nerve capped, sponge graft. There is nothing more said about it on my book, but my recollection of the case is that it was bleeding. Please handle this specimen carefully—I prize it very highly.

I send this simple statement of my reasons for adopting this treatment and my experience with it, indulging the hope that it may prove as great a comfort to many other members of the profession as it has to me, and that it may yet become a blessing to mankind.—*Southern Dental Journal.*

